# The Rapid and Long-Lasting Antidepressant Effects of Iridoid Fraction in *Gardenia Jasminoides* J.Ellis Are Dependent on Activating PKA-CREB Signaling Pathway

**DOI:** 10.3389/fphar.2022.896628

**Published:** 2022-06-08

**Authors:** Li Ren, Hailou Zhang, Weiwei Tao, Yin Chen, Zhilu Zou, XiaoYan Guo, Qinqin Shen, Quansheng Feng, Jingqing Hu

**Affiliations:** ^1^ School of Basic Medical Sciences, Chengdu University of Traditional Chinese Medicine, Chengdu, China; ^2^ Interdisciplinary Institute for Personalized Medicine in Brain Disorders and School of Chinese Medicine, Jinan University, Guangzhou, China; ^3^ Basic Teaching and Research Department of Integrated Chinese and Western Medicine, College of Traditional Chinese Medicine, Nanjing University of Chinese Medicine, Nanjing, China; ^4^ Institute of Basic Theory for Chinese Medicine, China Academy of Chinese Medical Sciences, Beijing, China

**Keywords:** *Gardenia jasminoides* Ellis, iridoid fraction, rapid and enduring antidepressant, PKA, CREB, BDNF

## Abstract

Lag periods of therapeutic efficacy cause poor compliance of patients, which has made solutions for rapid antidepressants the most urgent need in the depression study field at present. We have identified through our previous studies the rapid antidepressant effects of the traditional herb *Gardenia jasminoides* J.Ellis [Rubiaceae] (GJ) and its standardized fractions. Through screening different fractions of GJ, we decided to place our focus on the iridoid fraction of GJ (GJ-IF).

**Methods:** 1. Tail suspension test (TST), forced swimming test (FST), and novelty suppressed-feeding test (NSFT) were performed in sequence on mice after GJ-IF administration. 2. Mice in the model group were under chronic unpredictable mild stress (CUMS) for 3 w. After GJ-IF treatment, mice were placed in an open field test (OFT), Sucrose preference test (SPT), NSFT, TST, and FST. 3. Western Blot was performed to examine the expression of brain-derived neurotrophic factor (BDNF), Synapsin 1, cyclic-AMP dependent protein kinase A (PKA), phosphorylated cyclic-AMP responsive element-binding protein (p-CREB), and cAMP response element-binding protein (CREB). 4. Mice in the test group were administrated with GJ-IF after intraperitoneal injection of PKA blocker H89.

**Results:** 1. GJ-IF treatment significantly reduced the immobility time of TST at 1 d and FST at 26 h. 2. GJ-IF reversed the deficits induced by 3 w CUMS in SPT, TST, FST, and NSFT at 1 d and 26 h. The antidepressant effects of a single dose of iridoid fraction could also last for at least 14 d. 3. The results of molecule studies suggested that a single dose of GJ-IF activated p-CREB at 2 h and the PKA-CREB pathway at 1 d. The expression of BDNF did not significantly change from 30 min to 1 d after GJ-IF administration. 4. Blockade of PKA-CREB signaling pathway reversed the antidepressant effects of GJ-IF at 1 d, but not 30 min and 2 h.

**Conclusion:** GJ-IF is the crucial component in the rapid antidepressant of GJ. Rapid and sustained antidepressant effects of GJ-IF were dependent on activating the PKA-CREB signaling pathway.

## Graphical Abstract

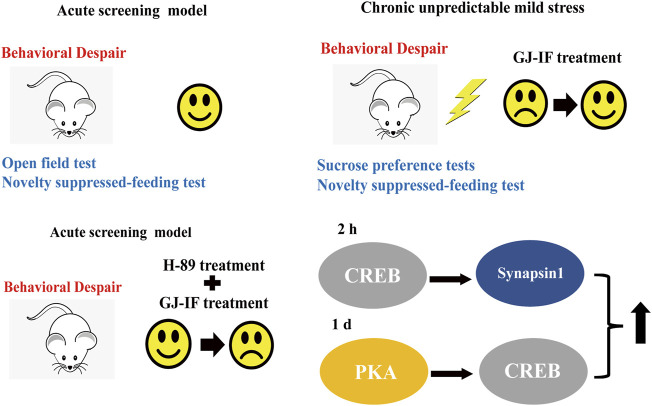

## Highlights

→GJ-IF showed rapid and enduring antidepressant effects in the acute drug screening model.
→GJ-IF reversed depressive behaviors induced by CUMS.
→GJ-IF exerted rapid and enduring antidepressant effects through the PKA-CREB pathway, but not BDNF.

## Introduction

Depression is a severe psychiatric disorder that affects an estimated 300 million people worldwide and has become the leading cause of mental health-related disease (Herrman et al., 2019). Conventional antidepressants such as selective serotonin reuptake inhibitors, although widely prescribed for the treatment of depression, are limited in their treatment effects due to their delayed onset of action (weeks to months) (Duman and Voleti, 2012). Therefore, fast-acting, efficacious therapies have become necessary to treat patients with depression. Recently, the first mechanistically new approach for the treatment of Major Depressive Disorder over the past 60 years, Spravato, a novel drug, has been approved as a nasal spray formulation to treat the depressive disorder (Krystal et al., 2020). The active ingredient of Spravato is esketamine, the s-isomer of ketamine. Randomized clinical trials have identified the efficacy of esketamine as a fast-acting antidepressant (Daly et al., 2018; Popova et al., 2019). However, the clinical application of esketamine triggered safety concerns about the increased risk of dissociation, abuse, suicidal ideation, and other adverse effects (Gastaldon et al., 2021; Vieira et al., 2021; Yang et al., 2021). Other disadvantages of esketamine treatment include stringent restrictions on medical indications and the requirement for close supervision under a doctor for its administration (Kasper et al., 2020). These problems with esketamine have substantially limited its clinical application. Thus, it is urgent to find a rapid-acting antidepressant drug that is safer and more effective.

Our previous studies have shown that *Gardenia jasminoides* J.Ellis [Rubiaceae] (GJ), a traditional herb that has been widely used in China, exhibits rapid effects highly similar to an antidepressant in acute and chronic mice models (Zhang et al., 2015). To find the main component of the rapid antidepressant effect in GJ, we performed behavioral drug screening on four standardized fractions of GJ. We found that petroleum ether of *Gardenia jasminoides* J.Ellis [Rubiaceae] (GJ-PE) and n-butyl alcohol fraction of *Gardenia jasminoides* J.Ellis [Rubiaceae] (GJ-BO) exhibited rapid antidepressant-like effects in acute and chronic mice models (Ren et al., 2016). In screening different fractions in both GJ-PE and GJ-BO, we focused on the iridoid fraction of GJ-BO, which, as observed, showed a better rapid antidepressant potent among other fractions. Iridoid fraction, which consists of geniposide, genipin-1-beta-gentiobioside, and three known bioactive constituents, is one of the most enriched ingredients of GJ (Chen et al., 2020). The medical effects of iridoid fraction of *Gardenia jasminoides* J.Ellis [Rubiaceae] (GJ-IF) are reported in studies of mellitus, arthritis, carotid artery thrombosis, and other diseases (Wang et al., 2013; Guo et al., 2014; Zhang et al., 2017; Hu et al., 2019). However, among these studies, few references have been made to the rapid antidepressant effects of GJ-IF.

Typical antidepressants increase synaptic plasticity in the hippocampus after a chronic administration. The rapid-acting antidepressant prototype drug, ketamine, can regulate novel synapse formation after administering a single dose (Li et al., 2010). BDNF, a member of the neurotrophin family, plays a vital role in regulating neuronal survival, differentiation, and synapse structure and function (Leal et al., 2015). Clinical and animal studies have demonstrated that BDNF is closely associated with depression disorder (Bjorkholm and Monteggia, 2016; Schroter et al., 2020). BDNF is one of the downstream target genes of CREB related to regulating synaptic plasticity (Chen et al., 2001). CREB is a cellular transcription factor that binds to certain DNA sequences called cAMP response elements (CRE) (Habener, 1990; Bourtchuladze et al., 1994). The transcription factor CREB is thought to play a role in the long-term effects of antidepressants, as it regulates the expression of many genes that have been implicated in depression and antidepressant response (Gundersen et al., 2013). PKA is the most well-studied cAMP-responsive protein kinase, which can directly phosphorylate CREB (Stern et al., 2011). PKA has essential effects on neuronal function and plasticity (Sanchez et al., 2004). Chronic treatment with selective serotonin reuptake inhibitor could activate both PKA and CREB in the hippocampus, and blockade of PKA-CREB signaling blunts the antidepressant effects of selective serotonin reuptake inhibitor (Thome et al., 2000; Hu et al., 2012). The PKA/CREB/BDNF pathway linked to neuronal functions, including protection of neurons and induction of synaptic plasticity, may involve the GJ-IF mechanism concerning the rapid and lasting effects of the antidepressant.

## Materials and Methods

### Animals

Male Kunming mice (20–25 g), aged 6–8 weeks (number: 1,102), were purchased from the China Academy of Military Medical Sciences (Beijing). Mice were habituated to animal facilities for 1 week before the experiment. The animals were maintained in a standard condition laboratory (12/12 h light/dark cycle, temperature 22 ± 2 °C, and room humidity, 50 ± 10%). Mice were fed a standard diet and filtered water. All the experimental procedures on animals conformed to the Guide for the Care and Use of Laboratory Animals and were approved by the Institutional Animal Care and Use Committee at the Chengdu University of Chinese Medicine.

### Drugs

**FIGURE 1:**
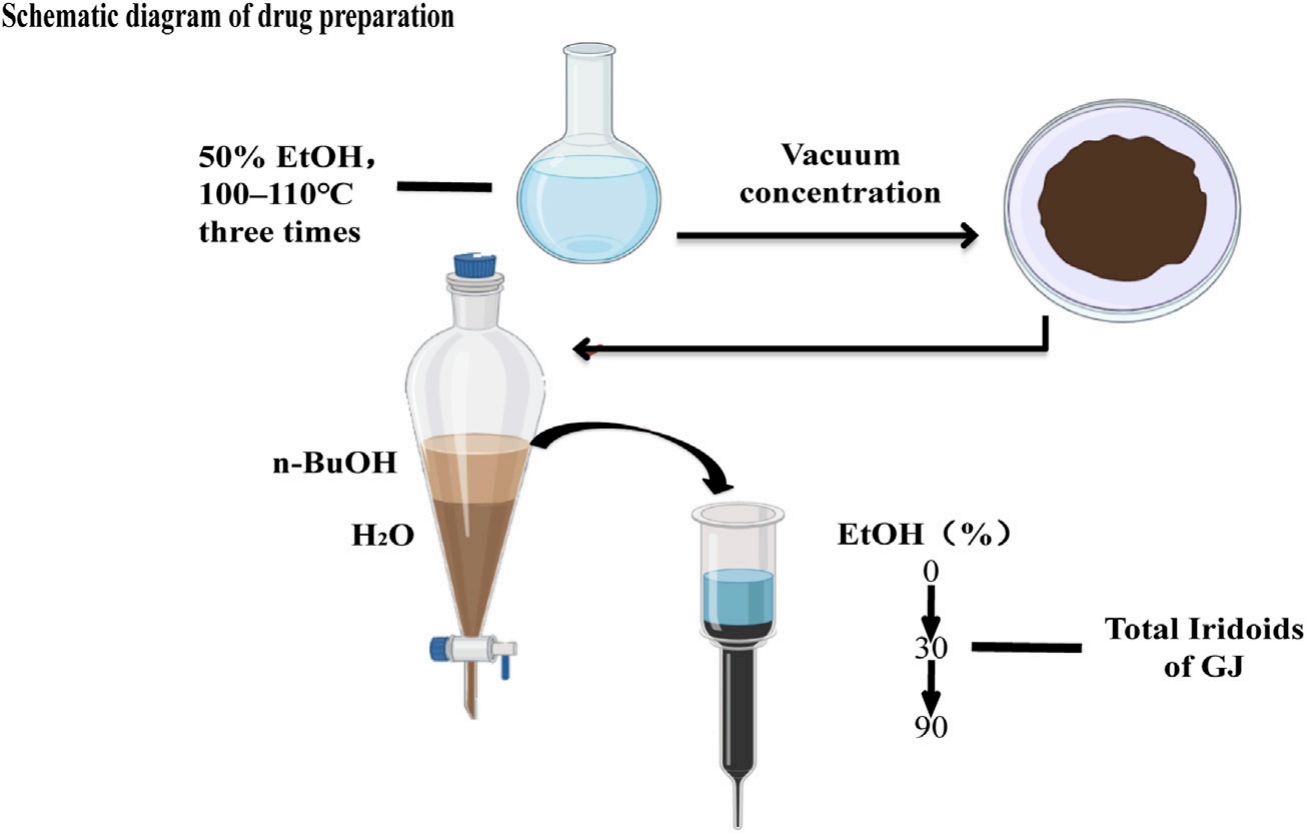
Schematic diagram of drug preparation of total iridoids of GJ.

The air-dried fruit of GJ was refluxed with 50% aqueous ethanol for 2 h at 100–110°C three times. The extract was concentrated in vacuo to afford a dark brown residue, dissolved in H_2_O, and partitioned with n-BuOH. The n-BuOH-soluble fraction was subjected to a macroporous resin column with a gradient (EtOH:H_2_O, 0:100→EtOH:H_2_O, 30:70 EtOH:H_2_O, 90:10) to yield three fractions (Fr.1–Fr.3). The 30% (v/v) EtOH eluate was the total iridoids of GJ (Figure 1). Mice can be treated with a single dose of GJ-IF intragastric administration. H-89 at 10 mg/kg (Sigma, St. Louis, MO, United States) was dissolved in 0.5% DMSO (0.06 umol/ml, dimethyl sulfoxide) and distilled water and were given an intraperitoneal injection at 30 min before iridoid fraction or vehicle administration.

### Qualitative Analysis of Active Compounds in the Extracts of GJ-IF

**FIGURE 2:**
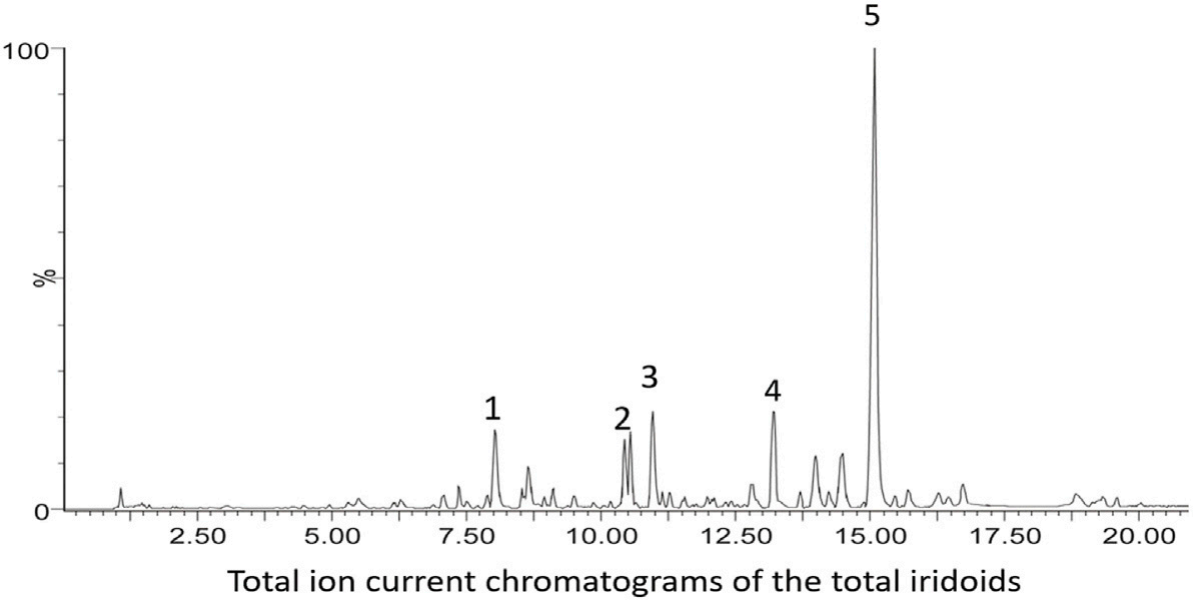
Quantitative analysis of GJ-IF. The UPLC chromatograms of GJ-IF are shown in Figure 2. The full MS and full MS/MS2 modes were generally used to capture all target ion information between MS1 and MS1,2 for qualitative analysis comparison. The major compounds in GJ-IF were identified to be geniposide, respectively.

UPLC was performed using a Waters ACQUITY UPLC system (Waters, Milford, MA, United States) equipped with a binary solvent delivery system and an auto-sampler. Chromatographic separation was performed on an ACQUITY UPLC HSS T3 column (2.1 mm × 100 mm, 1.7 m). The mobile phase was composed of A (water and 0.1% formic acid) and B (acetonitrile) under gradient elution conditions: 5%–20% B from 0 to 6 min, 20%–25% B from 6 to 15 min, and 25%–100% B from 15 to 20 min. The flow rate of the mobile phase was 0.4 ml/min, and the column temperature was maintained at 35°C. The injection volume was 2 μl, and the column eluent directly flowed into a mass spectrometer. The analyses were operated using MassLynxTM XS Software (Figure 2).

### Behavioral Tests

**FIGURE 3:**
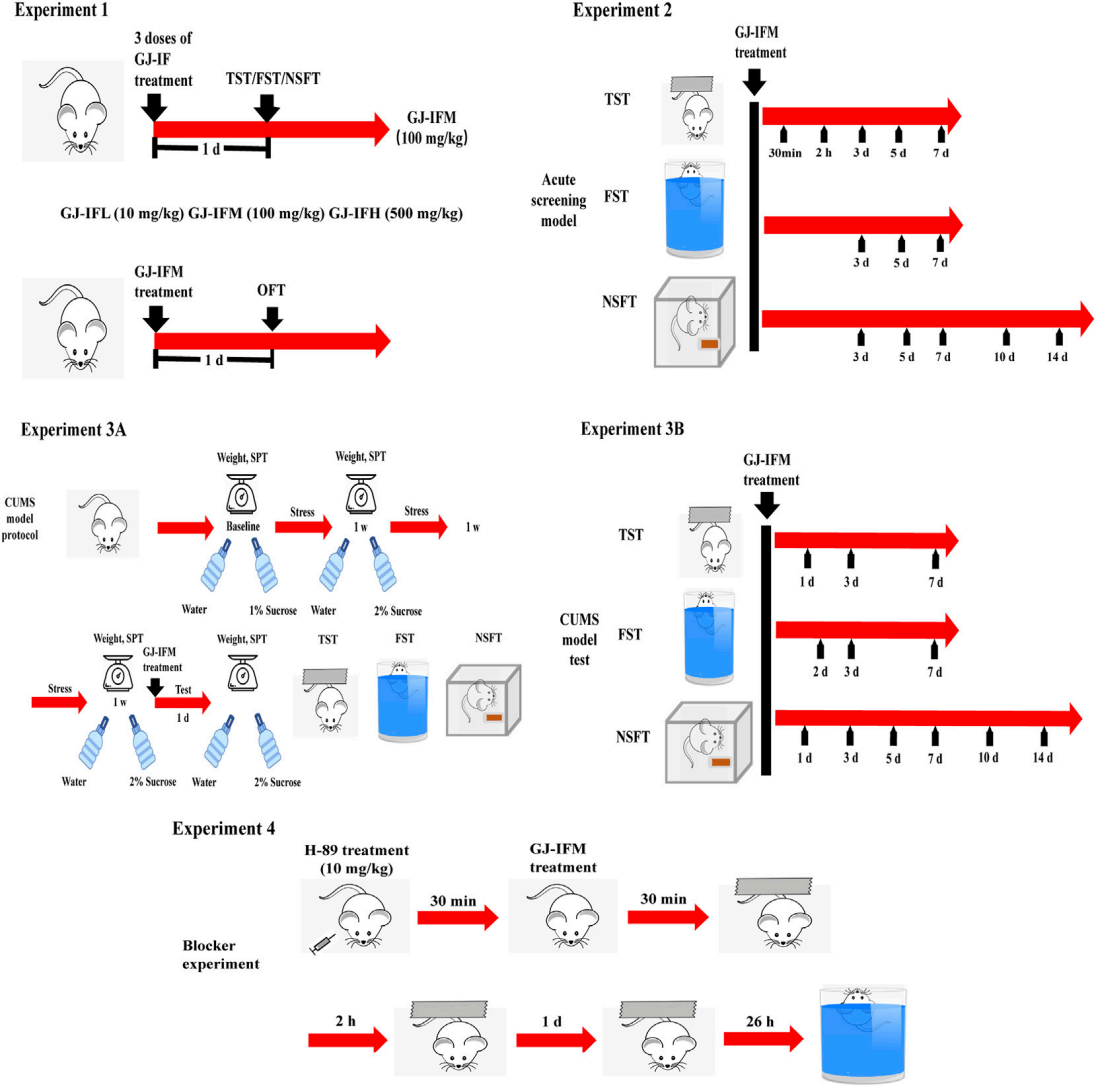
Experimental timeline and design of behavioral experiment.

Behavioral experiments were performed in the following order: experiment 1, experiment 2, experiment 3, and experiment 4. All tests were performed in 12 h light period (7:00–19:00) in a quiet room. Before testing, mice were put into the test room 2–3 h for environmental adaptation. Figure 3 outlined the experimental design and timing of behavioral experiments.

### Open Field Test

The OFT was used to assess anxiety-like behavior. In this test, spontaneous locomotor activity was measured in a computerized square arena device (40 × 40 × 15 cm) to monitor horizontal activity, including total distance traveled and time spent in the center zone. Mice were tested in a well-illuminated (∼300 lux) transparent acrylic cage for 5 min. The locomotion of mice was tracked in the two compartments near the bulkhead and central regions. The total distance (cm) and time spent in the central zone were analyzed by DigBehv software (Shanghai Jiliang, JLBehv).

### Tail Suspension Test

The TST is a widely used acute screening drug model. We performed the TST according to our previously reported methods (Ren et al., 2016). TST was performed in a computerized device, allowing four mice to be tested at one time. Each mouse was suspended 50 cm above the floor by adhesive tape placed approximately 1 cm from the tip of the tail. Mice were considered immobile when they were completely motionless after the first adaption phase. The immobility time of the last 4 min in a 6 min testing time was analyzed by DigBehv software (Shanghai Jiliang, JLBehv).

### Forced Swimming Test

The FST is also one of the most frequently used behavioral tests in preclinical antidepressant testing. We performed the FST following our previously reported protocol (Ren et al., 2016). Mice were removed from their home cages, placed individually into a transparent glass tank (40 cm high and 20 cm in diameter) filled with 30 cm of water (22°C–23°C), and allowed to swim for 6 min (Ren et al., 2016). The mice were considered immobile when floating in the water or making only small movements to keep their body in the balance after the first adaption phase. The total immobility time during the last 4 min of the 6 min testing period was analyzed by DigBehv software (Shanghai Jiliang, JLBehv).

### Novelty Suppressed-Feeding Test

The NSFT was performed according to our previously reported protocol (Ren et al., 2016). In the NSFT, mice were food-deprived for 1 d and then placed into a novel home cage without bedding. A single food pellet was placed in the center of the novel home cage. Each animal was placed at a corner of the open field at the beginning and allowed to explore freely in the arena for 10 min. The latency to begin eating, defined as active chewing of the pellet, was recorded. The amount of food consumption in the arena was also measured after the test as a control measure for appetite.

### Sucrose Preference Tests

The SPT procedure followed our published protocol (Ren et al., 2016). Sucrose preference and body weight were tested prior to assigning the mice to different groups and monitored weekly. Mice were individually housed and acclimatized to the two-bottle choice condition for 3 d, one filled with 1% sucrose solution and the other with filter water, followed by 1 d of water deprivation and a 2 h exposure to two identical bottles. The volume of sucrose solution and filter water was measured after 2 h exposure. Sucrose preference was defined as the ratio of the volume of sucrose versus total volume (sucrose + water) consumed during the 2 h test.

### Chronic Unpredictable Mild Stress Paradigm

The screened drug displaying rapid antidepressant potential was further tested on mice exposed to CUMS for optimal dose. The CUMS protocol followed our previously published methods (Ren et al., 2016). All CUMS mice were caged separately till the end of the experiment and received 3 w of unpredictable mild stress. Control mice were the group housed without stress administration. One of the following stressors was administered daily in a random and unpredictable order: food and water deprivation for 1 d, 45° tilt of cage for 1 d, cage shaking (horizontal shakes at high speed, 200 rpm) for 40 min, restraint in a 50 ml tube for 6 h, overnight illumination for 12 h, and soiled cage (200 ml water in the sawdust bedding) for 20 h. Three groups of mice separately received a battery of behaviors in the order of SPT, TST, FST, and NSFT. Sucrose preference and body weights were measured on days 1, 8, 15, and 21 during the paradigm.

### Western Blot

After the behavior experiments, mice were sacrificed immediately. The whole hippocampus (ventral and dorsal) was lysed in RIPA buffer containing protease inhibitors and phosphatase inhibitors. Protein concentration was determined colorimetrically by BCA assay (Pierce, Rockford, IL, United States). Protein lysates were separated by 10% SDS-PAGE (PKA, p-CREB, CREB), 15% SDS-PAGE (BDNF), and 8% SDS-PAGE (synapsin 1) electrophoresis and were transferred onto polyvinylidene difluoride (PVDF) membranes. After blocking with 1% BSA for 1 h, the membranes were differentially incubated with PKA (Cell Signaling Technology, #4782, 1:1,000), p-CREB (Cell Signaling Technology, #9198, 1; 500), CREB (Cell Signaling Technology, #9197, 1:1,000), BDNF (Santa Cruz Biotechnology, sc-546, 1:200), synapsin 1 (Cell Signaling Technology, #5297, 1:1,000), and β-tubulin (Epitomics, 1,879–1, 1:2,000) antibodies at room temperature (26 °C) for 1 h. Membranes were then washed three times (10 min/wash) with TBST, followed by incubation with horseradish peroxidase-conjugated secondary antibodies for 1 h. The membranes were washed three times with TBST (10 min/wash). The blots were visualized using the Immobilon Western Chemiluminescent HRP Substrate (MILLIPORE. WBKLS0500). PKA, p-CREB, CREB, BDNF, and synapsin 1 were normalized to β-tubulin. All experiments were performed three times.

### Statistical Analysis

Data are expressed as means ± SEM. Data analysis between two groups was analyzed using an unpaired Student’s *t*-test. Data analysis among three or more groups was conducted by one-way ANOVA with Dunnett’s multiple comparison or Tukey’s multiple comparison tests for independent measurement or repeated ANOVA for repeated measurement. The statistical analyses were performed using GraphPad Prism 8.0.2. Differences were considered to be significant at *p* < 0.05.

## Results

### Quantitative Analysis of GJ-IF Showed That Five Major Compounds Existed in GJ-IF

The total ion current chromatograms of the iridoids included five major compounds: shanzhiside, geniposidic acid, scandoside methylester, genipin-1-β-D-gentiobioside, and geniposide (Figure 2; Table 1).

**TABLE 1:** Quantitative analysis indicated five active compounds in the GJ-IF.

NO	Compounds	tR (time)	Formula	ESI(-) (m/z)	Fragment	Percentage (mg/g)
1	Shanzhiside	7.62	C16H24O11	391	229,185,121,109	32.33
2	Geniposidic acid	10.48	C16H22O10	373	211,149,123,105	28.05
3	Scandoside methylester	10.95	C17H24O11	449	241,193,139,101	38.97
4	Genipin-1-beta-gentiobioside	13.20	C23H34O15	549	225,207,123	48.79
5	Geniposide	15.08	C17H24O10	433	225,207,123,101	427.98

### GJ-IF at 100 mg/kg Showed a Rapid Antidepressant Effect in the Acute Screening Drug Model

**FIGURE 4:**
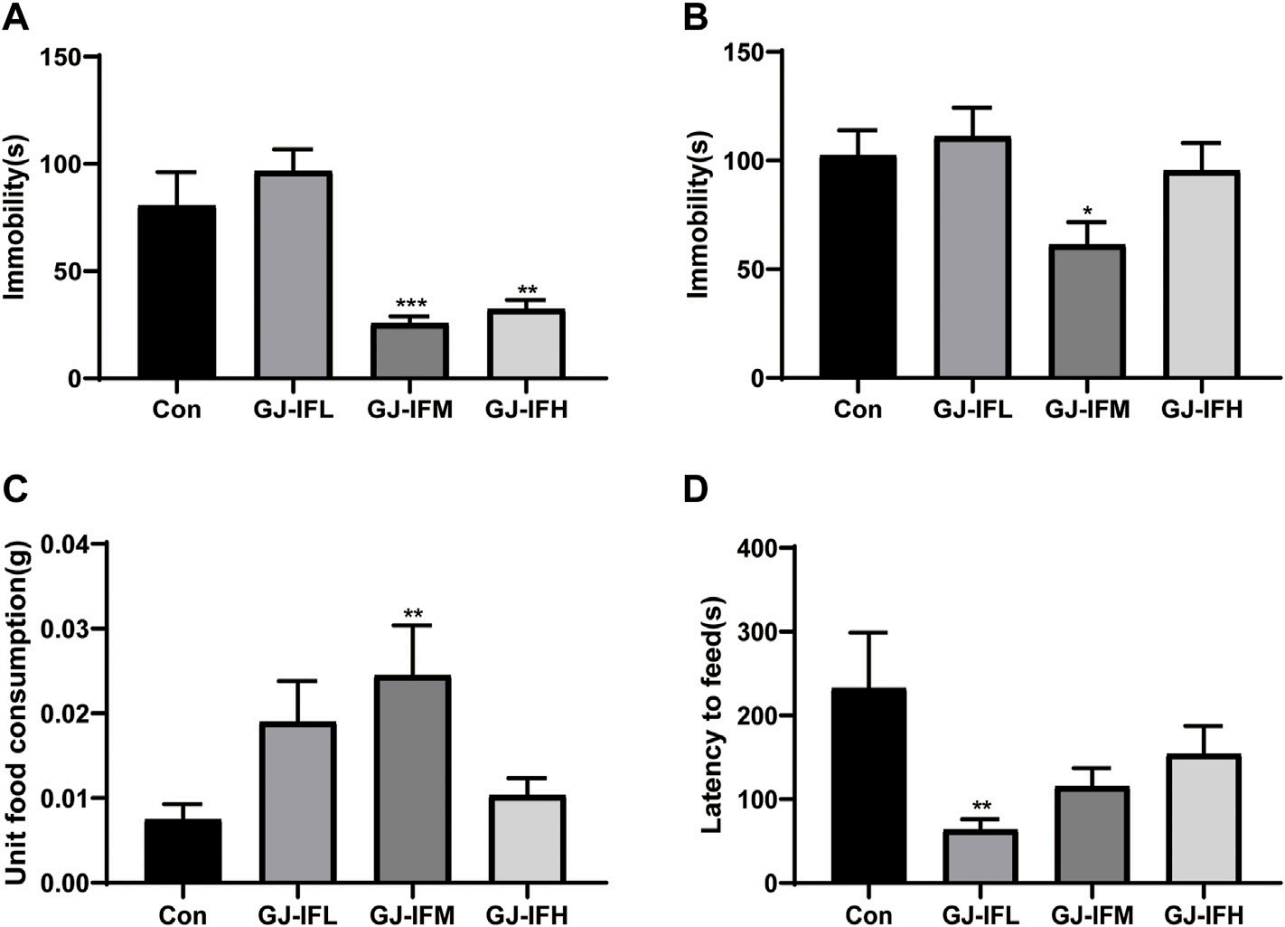
Screen for optimal dosage of GJ-IF with rapid antidepressant potential. The low, medium, and high doses of GJ-IF are abbreviated as GJ-IFL, GJ-IFM, GJ-IFH. **(A)** Tail suspension test at 1 d after administration of GJ-IF. Immobility time was measured for the last 4 min during the 6 min testing time. One-way ANOVA, ^**^ *p* < 0.001, ^***^ *p* < 0.001; control *vs.* GJ-IF. **(B)** Forced swim test at 1 d after administration of three doses of GJ-IF. One-way ANOVA, ^*^ *p* < 0.05; control *vs*. GJ-IF. **(C)** Unit food consumption during 10 min test of novelty suppressed-feeding at 1 d after administration of three doses of GJ-IF. One-way ANOVA, ^**^ *p* < 0.01; control *vs*. GJ-IF. **(D)** Time of latency to eat during 10 min test of novelty suppressed-feeding at 1 d after administration of three doses of GJ-IF. One-way ANOVA, ^**^ *p* < 0.01; control *vs*. GJ-IF.

The latency to feed in the NSFT test has been to index anxiety-like behaviors (Santarelli et al., 2003). A range doses (10, 100, and 500 mg/kg) of GJ-IF were tested at 1 d in TST (one-way ANOVA, GJ-IFM: *p* < 0.001; *n* = 9–12/group.) (Figure 4A), 1 d FST (one-way ANOVA, GJ-IFM: *p* < 0.05; *n* = 10–13/group.) (Figure 4B), and 1 d NSFT (unit food consumption: one-way ANOVA, GJ-IFM: *p* < 0.01; *n* = 9–11/group; latency to feed: one-way ANOVA, GJ-IFM: *p* > 0.05; *n* = 7–10/group.) (Figures 4C,D). The optimal dose was 100 mg/kg.

### GJ-IFM Treatment Did Not Have Effects on Locomotion

**FIGURE 5:**
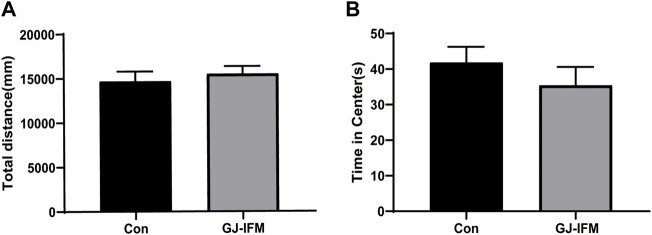
The effects of a single dose of GJ-IFM on OFT at 1 d in KM mice. **(A)** Total distance traveled was not affected by the treatment. *t*-test, *p* > 0.05; control *vs*. GJ-IFM. **(B)** Time spent on the central zone was not affected by the treatment *t*-test, *p* > 0.05; control *vs*. GJ-IFM.

There was no impact on locomotion (*t*-test, *p* > 0.05, *n* = 10/group.) (Figure 5A) or time spent in the central zone (*t*-test, *p* > 0.05, *n* = 10/group) (Figure 5B) 30 min after a single dose of the GJ-IFM (100 mg/kg) treatment.

### The Antidepressant Action of GJ-IFM Could Last for 2 Weeks

**FIGURE 6:**
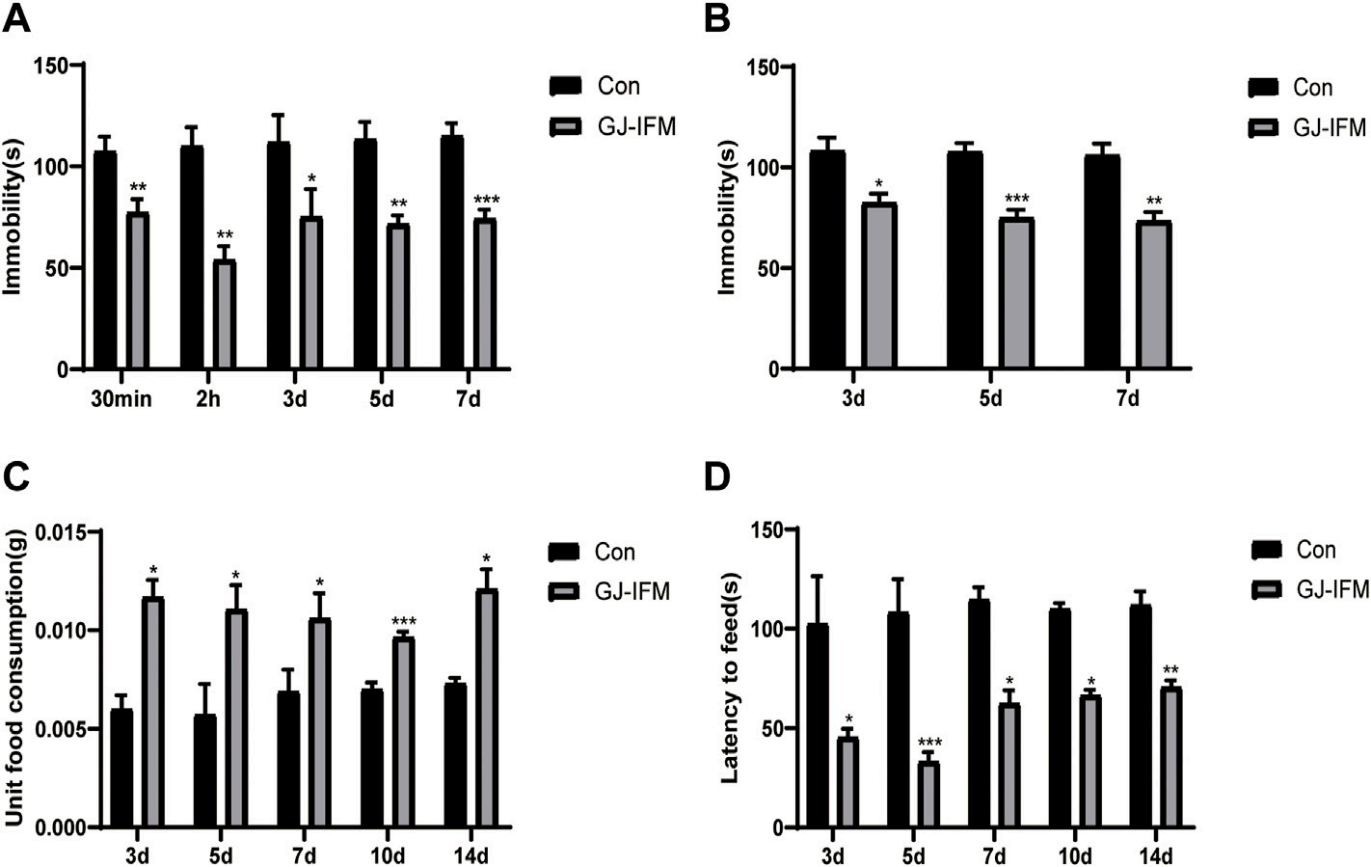
The length of time for enduring rapid antidepressant effects of GJ-IFM on TST, FST, NSFT. **(A)** Tail suspension test after administration of GJ-IFM. *t*-test, ^*^ *p* < 0.05, ^**^ *p* < 0.01, ^***^ *p* < 0.001; control *vs*. GJ-IFM. **(B)** Forced swim test after administration of GJ-IFM. *t*-test, ^*^ *p* < 0.05, ^**^ *p* < 0.01, ^***^ *p* < 0.001; control *vs*. GJ-IFM. **(C)** Unit food consumption during 10 min test of novelty suppressed-feeding at 1 d after administration of three doses of GJ-IFM. ^*^ *p* < 0.05, ^***^ *p* < 0.001; control *vs.* GJ-IFM. **(D)** Time of latency to eat during 10 min test of novelty suppressed-feeding at 1 d after administration of three doses of GJ-IFM. *t*-test, ^*^ *p* < 0.05, ^**^ *p* < 0.01, ^***^ *p* < 0.001; control *vs*. GJ-IFM.

The GJ-IFM at a single dose of 100 mg/kg treatment provided instant induction of antidepressant effect in terms of significant reduction of immobile time in TST at 30 min, and this effect remained persistent at 2 h and 3, 5, and 7 d (TST: *t*-test, 30 min: *p* < 0.01, *n* = 10/group; 2 h: *p* < 0.01, *n* = 9/group; 3 d: *p* < 0.05, *n* = 8–9/group; 5 d: *p* < 0.01, *n* = 5–10/group; 7 d: *p* < 0.001, *n* = 4–10/group) (Figure 6A). Similarly, the effect was detected in the FST at 3, 5, and 7 d (FST: *t*-test, 3 d: *p* < 0.05, *n* = 5–10/group; 5 d: *p* < 0.001, *n* = 6–11/group; 7 d: *p* < 0.01, *n* = 4–10/group) (Figure 6B), despite it did not last for 14 d. In NSFT tested at 3, 5, 7, 10, and 14 d after a single dose of GJ-IFM (100 mg/kg) treatment, there was a significant increase in unit food consumption (unit food consumption: *t*-test, 3 d: *p* < 0.05, *n* = 10–13/group; 5 d: *p* < 0.05, *n* = 8–9/group; 7 d: *p* < 0.05, *n* = 9/group; 10 d: *p* < 0.001, *n* = 4–10/group; 14 d: *p* < 0.05, *n* = 4–10/group.) (Figure 6C) and a decrease in latency to feed (latency to feed: *t*-test, 3 d: *p* < 0.05, *n* = 10–11/group; 5 d: *p* < 0.001, *n* = 8–9/group; 7 d: *p* < 0.05, *n* = 7–8/group; 10 d: *p* < 0.05, *n* = 4–10/group; 14 d: *p* < 0.01, *n* = 4–10/group.) (Figure 6D).

### GJ-IFM Showed Fast-Onset Antidepressant Effects in CUMS Model

**FIGURE 7:**
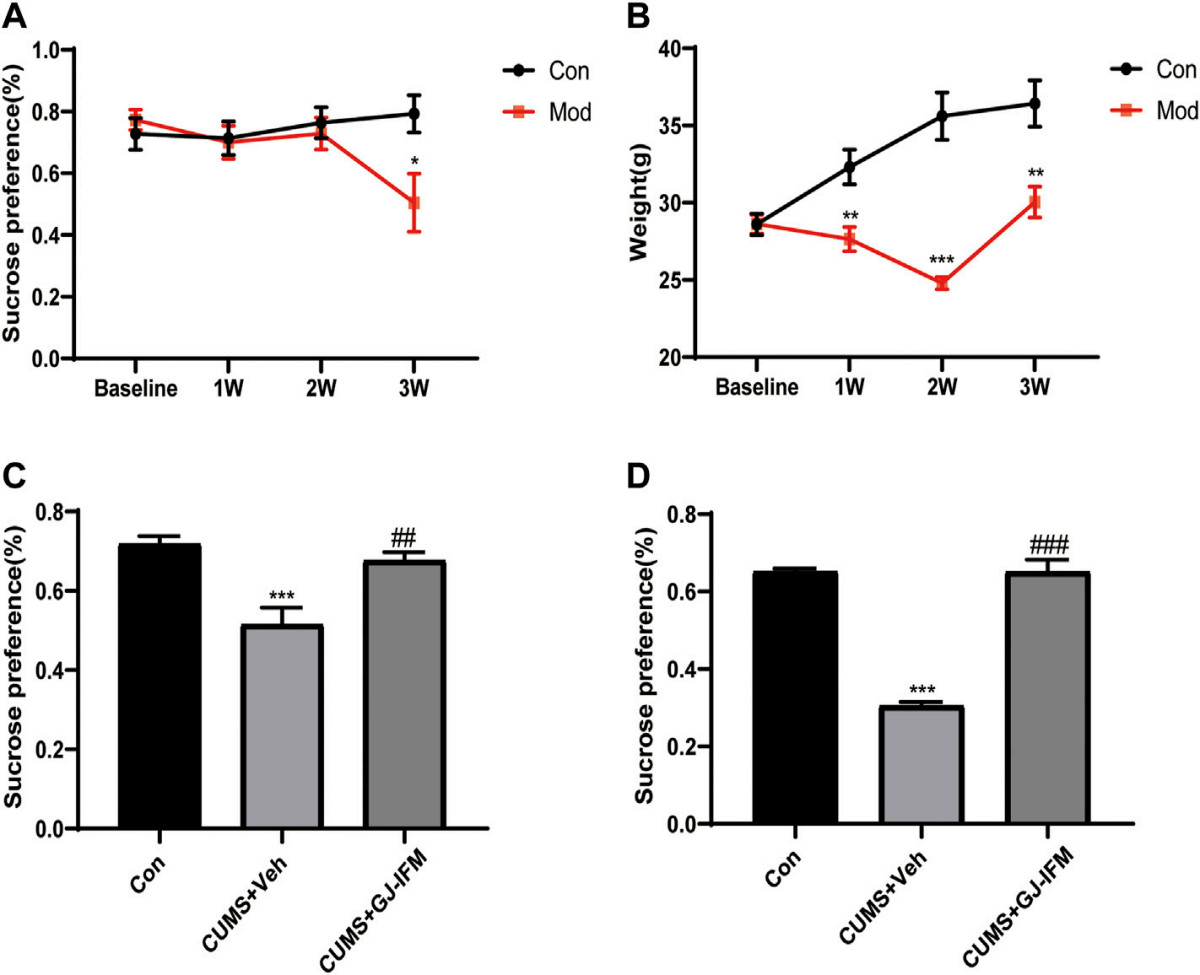
The effects of a single dose of GJ-IFM (100 mg/kg) in CUMS mice on SPT and body weight. **(A)** SPT after 3 weeks of CUMS administration. *t*-test, ^*^ *p* < 0.05; control *vs*. CUMS + vehicle. **(B)** Body weight after 3 weeks of CUMS administration. *t*-test, ^**^ *p* < 0.01, ^***^ *p* < 0.001; control *vs*. CUMS + vehicle. **(C)** The effects of a single dose of GJ-IFM (100 mg/kg) treatment in CUMS mice on SPT at 1 d. One-way ANOVA, ^***^ *p* < 0.001; control *vs*. CUMS + vehicle; ^##^ *p* < 0.01; CUMS + vehicle *vs*. CUMS + GJ-IFM. **(D)** The effects of a single dose of GJ-IFM (100 mg/kg) treatment in CUMS mice on SPT on 10 d. One-way ANOVA, ^***^ *p* < 0.001, control *vs*. CUMS + vehicle; ^###^ *p* < 0.001, CUMS + vehicle *vs*. CUMS + GJ-IFM.

**FIGURE 8:**
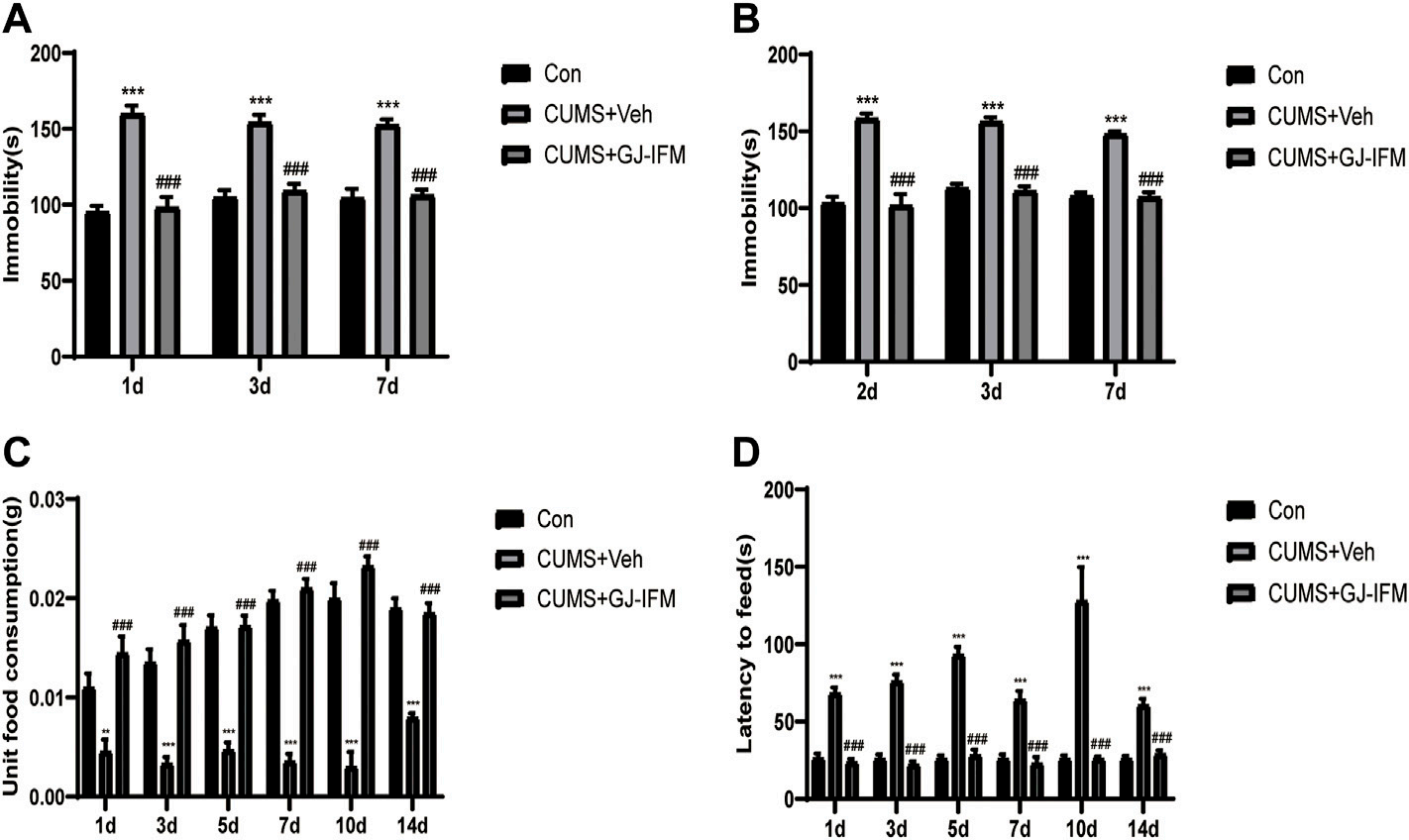
The enduring effects of a single dose of GJ-IFM (100 mg/kg) treatment on TST, FST, NSFT in CUMS mice. **(A)** A significant decrease in immobility time in TST from 1 to 7 d was observed in the mice treated with GJ-IFM. One-way ANOVA, ^***^ *p* < 0.001, control *vs*. CUMS + vehicle. ^###^ *p* < 0.001, CUMS + vehicle *vs*. CUMS + GJ-IFM. **(B)** CUMS mice significantly increased immobility time in the FST. GJ-IFM significantly reduced prolonged immobility time induced by CUMS from 2 to 7 d. One-way ANOVA, ^***^ *p* < 0.001, control *vs*. CUMS + vehicle. ^###^ *p* < 0.001, CUMS + vehicle *vs*. CUMS + GJ-IFM. **(C)** GJ-IF significantly decreased unit food consumption during 10 min test of NSF from 1 to 14 d. One-way ANOVA, ^**^ *p* < 0.01, ^***^ *p* < 0.001, control *vs*. CUMS + vehicle. ^###^ *p* < 0.001, CUMS + vehicle *vs*. CUMS + GJ-IFM. **(D)** GJ-IF significantly decreased the time of latency to eat during 10 min test of NSFT from 1 to 14 d. One-way ANOVA, ^***^ *p* < 0.001, control *vs*. CUMS + vehicle. ^###^ *p* < 0.001, CUMS + vehicle *vs*. CUMS + GJ-IFM.

**FIGURE 9:**
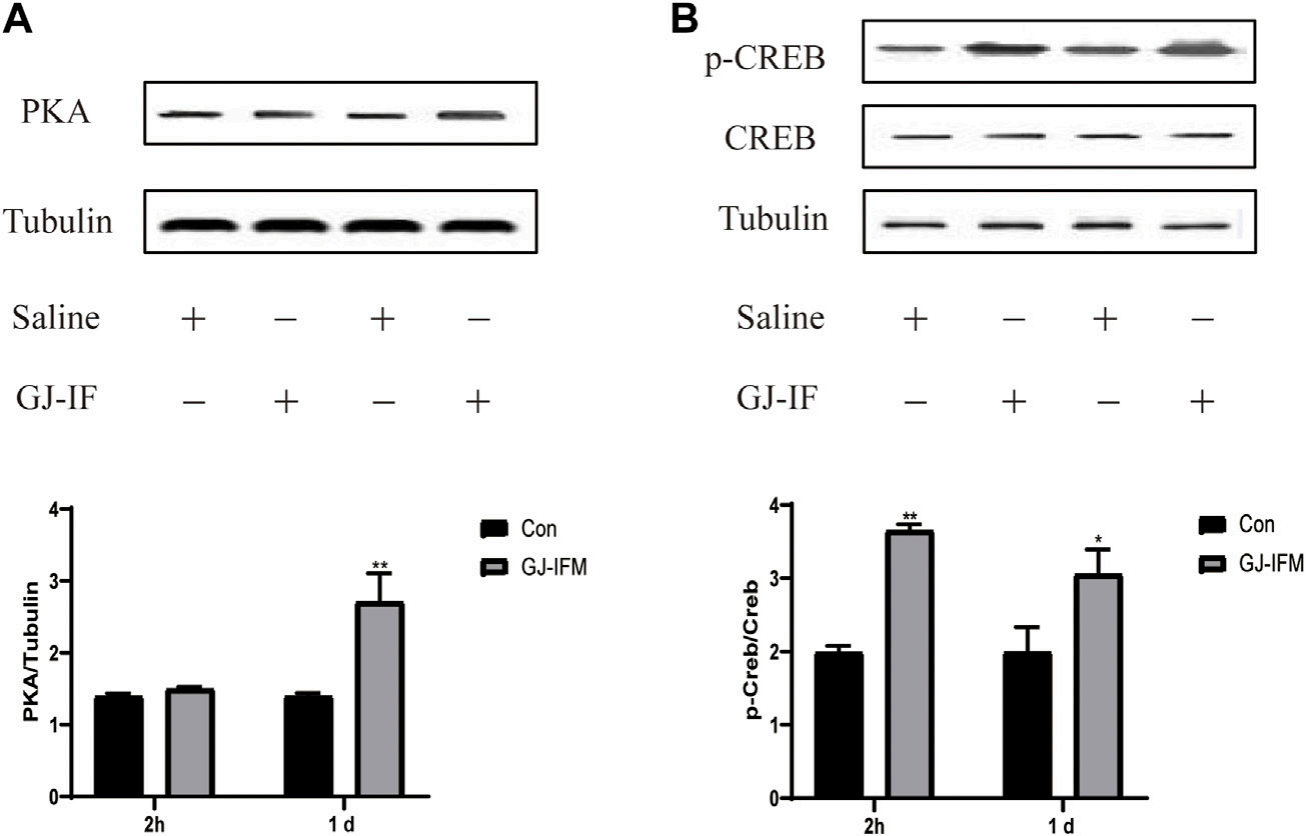
p-CREB activated at 2 h and 1 d after GJ-IFM treatment, while PKA activated at 1 d. **(A)** The expression of PKA significantly enhanced in the hippocampus at 1 d after GJ-IFM administration, but not 2 h. *t*-test, 2 h: *p* > 0.05, 1 d: ^**^ *p* < 0.01; control *vs*. GJ-IFM. **(B)** The expression of p-CREB significantly enhanced in the hippocampus at 2 h and 1 d after GJ-IF administration. *t*-test, 2 h: ^**^ *p* < 0.01, 1 d: ^*^ *p* < 0.05; control *vs*. GJ-IFM.

**FIGURE 10:**
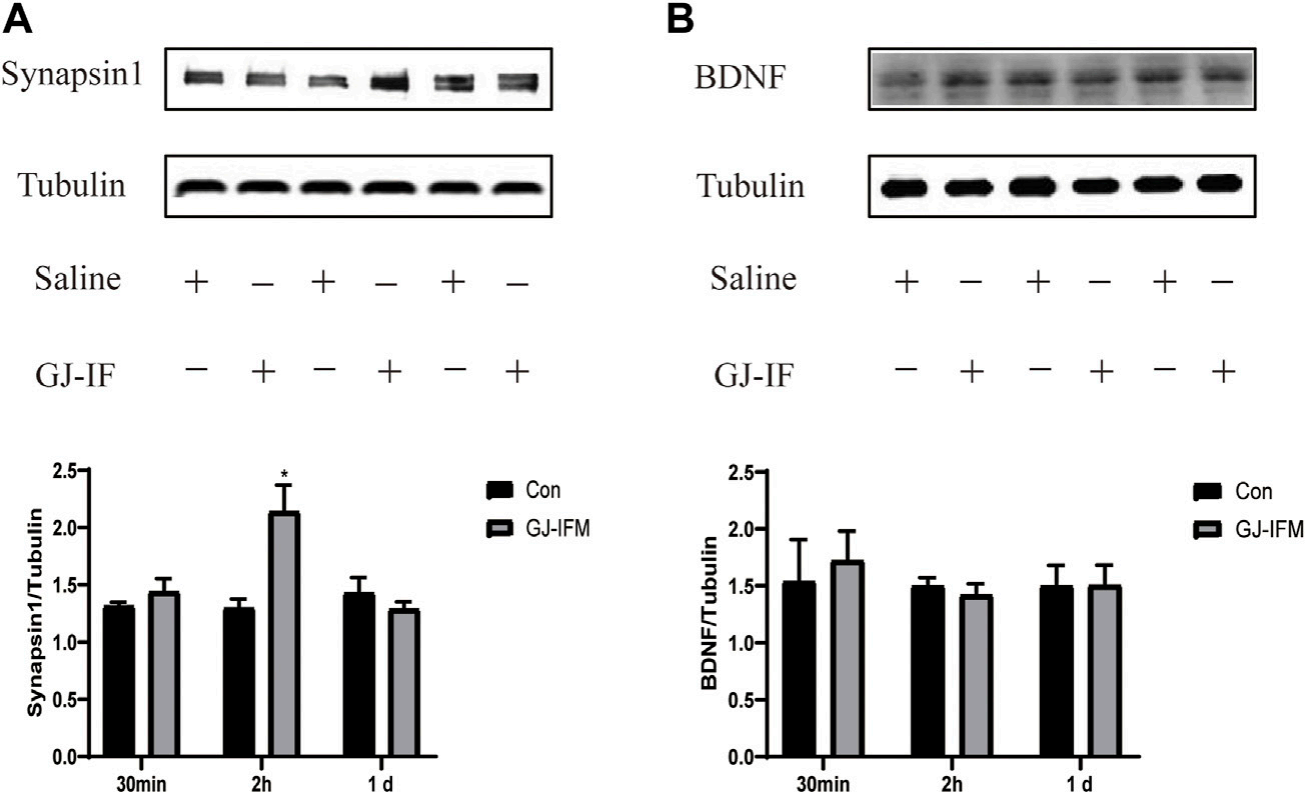
GJ-IFM drastically enhanced expression of synaptic protein synapsin 1 but not BDNF. **(A)** The expression of synapsin 1 increased at 2 h after GJ-IFM administration; however, there was no significant change at 30 min and 1 d. *t*-test, 30 min: *p* > 0.05, 2 h: ^*^ *p* < 0.05, 1 d: *p* > 0.05; control *vs*. GJ-IFM. **(B)** There was no significant escalation in BDNF protein expression at 30 min, 2 h, and 1 d. *t*-test, 30 min: *p* > 0.05, 2 h: *p* > 0.05, 1 d: *p* > 0.05; control *vs*. GJ-IFM.

In our study, we found that CUMS mice exhibited a significant decrease in sucrose preference after 3 w (*t*-test, 3 w: *p* < 0.05, *n* = 4–11/group) (Figure 7A). Losing body weight was another indicator of depression induced by CUMS. The body weight of CUMS mice significantly decreased from 1 to 3 weeks after a series of stresses (*t*-test, 3 w: *p* < 0.01, *n* = 9–15/group) (Figure 7B). A single dose of GJ-IFM treatment significantly increased sucrose preference from 1 d (one-way ANOVA, *p* < 0.01, *n* = 10/group.) (Figure 7C) to 10 d (one-way ANOVA, *p* < 0.001, *n* = 4–6/group) (Figure 7D).

The time-course of antidepressant action of GJ-IFM could last for 2 weeks in the CUMS model, which was consistent with the time-course of the acute model. GJ-IFM reduced the immobile time of TST at 1, 3, and 7 d and FST at 2, 3, and 7 d (TST: one-way ANOVA, 1 d, *p* < 0.001, *n* = 10/group; 3 d, *p* < 0.001, *n* = 6–7/group; 7 d, *p* < 0.001, *n* = 6–7/group; FST: one-way ANOVA, 2 d: *p* < 0.001, *n* = 10/group; 3 d: *p* < 0.001, *n* = 6–7/group; 7 d: *p* < 0.001, *n* = 6–7/group) (Figures 8A,B). In NSFT tested at 1, 3, 5, 7, 10, and 14 d that post a single dose of GJ-IFM treatment, the unit food consumption and latency to feed significantly increased (unit food consumption: one-way ANOVA, 1 d: *p* < 0.001, *n* = 9–10/group; 3 d: *p* < 0.001, *n* = 9–10/group; 5 d: *p* < 0.001, *n* = 10/group; 7 d: *p* < 0.001, *n* = 7–10/group; 10 d: *p* < 0.001, *n* = 4–6/group; 14 d: *p* < 0.001, *n* = 6–7/group; latency to feed: one-way ANOVA, 1 d: *p* < 0.001, *n* = 10/group; 3 d: *p* < 0.001, *n* = 7–10/group; 5 d: *p* < 0.001, *n* = 10/group; 7 d: *p* < 0.001, *n* = 7–10/group; 10 d, *p* < 0.001, *n* = 4–6/group; 14 d: *p* < 0.001, *n* = 6–7/group) (Figures 8C,D).

### PKA and CERB, but Not BDNF, Are Involved in GJ-IFM’s Fast and Enduring Antidepressant Effects

GJ-IFM had no influence on PKA expression at 2 h (PKA: *p* > 0.05) (Figure 9A), while it significantly increased the expression of p-CREB and synapsin 1 at 2 h (pCREB/CREB: *p* < 0.01; synapsin 1: *p* < 0.05) (Figures 9B,10A). The expression of PKA and p-CREB increased at after a single administration of GJ-IFM (PKA: *p* < 0.01; p-CREB/CREB: *p* < 0.05) (Figures 9A,B).

We did not find the activation of synapsin 1 at 1 d (*p* > 0.05) (Figure 10A). To identify the effective time point of GJ-IFM on synaptic plasticity, we tested the synapsin 1 expression at 30 min and found no significant change (*p* > 0.05) (Figure 10A). There was no change in BDNF expression at any time point (30 min, 2 h, 1 d) measured after GJ-IFM treatment (*p* > 0.05) (Figure 10B).

### Blockade of PKA-CREB Pathway Blunted Antidepressant Effects of GJ-IFM on TST at 1 d and FST at 26 h

**FIGURE 11:**
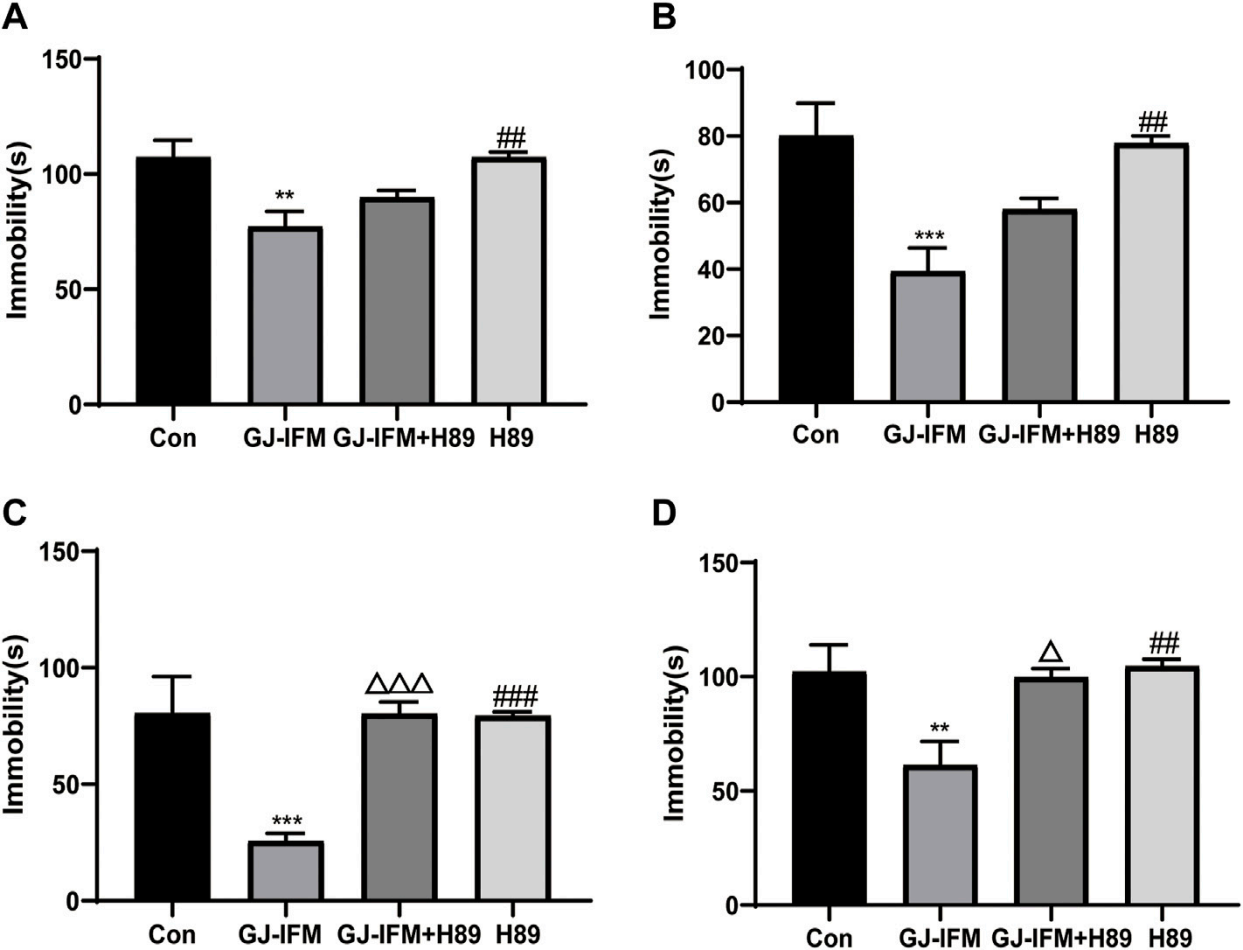
The effects of PKA antagonist H89 on the antidepressant response after a single GJ-IFM treatment. **(A)** The effect of H89 pretreatment on immobility time in TST 30 min after GJ-IFM treatment in KM mice. One-way ANOVA, *p* > 0.05, GJ-IFM *vs*. GJ-IFM + H89. **(B)** The effect of H89 pretreatment on immobility time in TST 2 h after GJ-IFM treatment in KM mice. One-way ANOVA, *p* > 0.05, GJ-IFM *vs*. GJ-IFM + H89. **(C)** The effect of H89 pretreatment on immobility time in TST 1 d after GJ-IFM treatment in KM mice. One-way ANOVA, ^△△△^ *p* < 0.001, GJ-IFM *vs*. GJ-IFM + H89. **(D)** The effect of H89 pretreatment on immobility time in FST at 26 h after GJ-IFM treatment in KM mice. One-way ANOVA, ^△^ *p* < 0.05, GJ-IFM *vs*. GJ-IFM + H89.

To test the involvement of the PKA-CREB pathway, we utilized a PKA-specific blocker H89. H89 did not block the fast-onset effects of GJ-IFM in TST at 30 min (one-way ANOVA, *p* > 0.05, *n* = 7–10/group) (Figure 11A) and 2 h (one-way ANOVA, *p* > 0.05, *n* = 7–10/group) (Figure 11B). But the rapid antidepressant action of GJ-IFM was blocked by H89 in TST at 1 d (one-way ANOVA, *p* < 0.001, *n* = 8–11/group.) (Figure 11C) and FST at 26 h (one-way ANOVA, *p* < 0.05, *n* = 8–13/group) (Figure 11D).

## Discussion

The discovery of ketamine as a rapid-acting antidepressant has been the most critical development in psychiatry for a few decades (Kraus et al., 2019). Ketamine is a non-competitive N-methyl-d-aspartic acid (NMDA) glutamate receptor antagonist (Berman et al., 2000; Zarate et al., 2006). (S)-Ketamine, the racemic S-enantiomer of ketamine, displays an approximately fourfold greater affinity for the glutamate NMDA receptor (NMDAR) than the other isomer, (R)-ketamine (Moaddel et al., 2013). As the NMDAR inhibition has been regarded as playing a vital role in mechanisms of rapid antidepressant effects, esketamine started being selected as a novel option to treat depression (Zhang and Hashimoto, 2019). However, both ketamine and esketamine had significant adverse events (Zhu et al., 2016; Molero et al., 2018; Iqbal and Mathew, 2020). In recent years, medicinal plants and natural products have been receiving increasing attention for their safety and high performance in medical treatment. GJ, a popular shrub in the Rubiaceae family, with its chemical constituents, has been proved to exert rapid antidepressant effects (Ren et al., 2016; Chen et al., 2020). Our goal is to find GJ’s most effective rapid antidepressant candidate component.

We aimed to investigate whether GJ-IF can exhibit rapid antidepressant effects through this study. TST and FST are two acute behavioral paradigms we used in our study for screening antidepressants. The immobility time in the two behaviors reflects the level of despair (Porsolt et al., 1978; Cryan et al., 2005). The NSFT procedure is a conflict test that elicits competing motivations: the drive to eat and the fear of venturing into the center of a brightly lit arena (Bodnoff et al., 1988). We screened three doses of GJ-IF and identified that the medium (100 mg/kg) dose of GJ-IF (GJ-IFM) had the most rapid antidepressant potential. GJ-IFM exerted significant fast-onset antidepressant effects in TST, FST, and NSFT after a single acute administration. Based on this, we continued to test the rapid antidepressant effects of GJ-IFM in CUMS, which is similar to the depression pathological state of the human being. CUMS is a widely used depression model which mimics a state of impaired reward observed in depressive disorder (Hill et al., 2012). A reduced sucrose preference in the depression model reflects anhedonia, a core symptom of depression (Burstein and Doron, 2018). Conventional antidepressants can reduce depression-like behaviors induced by CUMS after chronic repeated administration (Willner, 2005). Replicated observations have demonstrated that a single dose of ketamine can produce meaningful clinical improvement within hours (Krystal et al., 2013). Ketamine can rapidly reverse behavioral deficits caused by chronic stress exposure after a single dose of treatment (Li et al., 2011). Similar to ketamine, in our study, at 1 d after a single dose of administration, GJ-IFM significantly reversed the following aspects induced by the CUMS model, including decreased sucrose preference, lengthened immobility time in TST and FST, longer latency to feed, and less food consumption in NSFT induced by CUMS model. The enduring antidepressant effects of GJ-IF can last longer during the time course than those of both GJ and other components in GJ, such as oil from the fruit of GJ and Crocin (Tao et al., 2014; Tang et al., 2020), especially in NSFT test after CUMS. A higher dose is required for another enriched ingredient yellow pigment of GJ (GJY) to induce rapid antidepressant effects, and its antidepressant effects are not as enduring as iridoid fraction (Wu et al., 2016; Kasper et al., 2020). It has been reported that iridoid fraction can directly affect the central nervous system by passing through blood–brain barrier (Zhang et al., 2011). Therefore, GJ-IF is the most promising rapid antidepressant candidate constituent among GJ. It has been demonstrated that geniposide, the major compound in iridoids, possesses antidepressant effects (Cai et al., 2015; Wang et al., 2016). However, it failed to show the rapid antidepressant potential (Zhang et al., 2015), suggesting that other less enriched compounds or their interactions with geniposide may play critical roles, which is currently under our investigation.

Our previous research indicated that the expression of BDNF is related to the onset time point of GJ (Zhang et al., 2015). In this study, we found that GJ-IFM did not upregulate BDNF in the hippocampus of mice from 30 min to 1 d after a single dose of drug administration, and our finding is consistent with GJ-BO (Ren et al., 2016). A single dose of ketamine has been found to significantly increase levels of synaptic proteins, including synapsin 1 in the prefrontal cortex (PFC) from 2 h to 3 d after acute administration, which has demonstrated that synaptogenesis activity in the medial prefrontal cortex (mPFC) pyramidal neurons plays a crucial role in the rapid onset antidepressant effects of ketamine (Li et al., 2010). Studies concerning mechanisms of rapid antidepressant effects have been focused on synaptic plasticity changes in PFC but not the hippocampus. In our present study, we placed great attention on the synaptic plasticity change induced by CUMS and the synaptic plasticity involved in the mechanism of rapid antidepressant effects of GJ-IF in the hippocampus. The quick upregulation of synapsin 1 in the hippocampus occurred at 2 h, the time point when synapsin 1 was initially activated by ketamine, and subsequently returned to normal level at 1 d after a single acute administration of GJ-IFM. These results suggest that depression-related PFC and hippocampus circuits may overlap in some domains, such as quick regulation of antidepressant activities. Our study thus far has shown that GJ-IFM activated p-CREB at 2 h and PKA-CREB signaling at 1 d. In addition, PKA-blocker H89 blocked the rapid antidepressant effects of GJ-IFM at 1 d, which gave additional evidence of activating PKA. However, H89 did not affect GJ-IFM at 30 min and 2 h, indicating activation of p-CREB as a prerequisite for GJ-IFM’s initial effective antidepressant action of GJ-IFM and PKA-CREB signaling as a necessary condition for the sustained antidepressant action of GJ-IFM. Synapsin 1 in the hippocampus did not involve the sustained antidepressant effects of GJ-IFM. Whether other synaptic proteins such as NMDAR in the hippocampus are related to the lasting antidepressant action of GJ-IFM warrants further investigations.

We had made a hypothesis that GJ-IF activates PKA/CREB/BDNF to produce rapid antidepressant effects. Based on studies of CREB signaling, it has been proposed that PKA could be responsible for Ser-133 phosphorylation of CREB in response to hippocampal synaptic activity. As PKA can translocate to the nucleus and phosphorylating CREB at Ser-133, rises in Ca^2+^ could cause CREB phosphorylation through Ca^2+^-activated adenylyl cyclase and increases in PKA activity (Deisseroth et al., 1996). Evidence showed that activation of PKA may cause a late phase of long-term potentiation (Huang et al., 2006). Accumulating evidence also demonstrated the essential role of CREB in mediating hippocampal L-LTP (Wu et al., 2007). It was reported that a single induction of strong LTP produced long-lasting cytoskeletal reorganization characterized by an increase in F-actin content within dendritic spines in an animal *in vivo* (Tominaga-Yoshino et al., 2008). LTP *in vivo* can last a few weeks or even a few months. Hippocampal pCREB is particularly associated with protocols that induce stable LTP lasting months (Abraham et al., 2002). All the evidence suggests that the activation of the PKA/CREB pathway involved regulating the long-lasting antidepressant effects of GJ-IFM from 1 d to 2 w. However, other kinases such as Ca^2+^/CaM-dependent kinases I, II, and IV can also be activated by Ca^2+^/CaM and phosphorylate CREB on Ser-133 (Deisseroth et al., 1996). This explains that GJ-IFM treatment merely activated p-CREB at 2 h but not PKA. The neurotrophic hypothesis of depression proposes that decreased levels of BDNF in the hippocampus lead to the pathogenesis of depression, and treatment with antidepressants enhances BDNF levels and alleviates depressive symptoms (Rana et al., 2021). However, we did not find a significant change from 30 min to 1 d after GJ-IFM treatment. Other pathways may be involved in influencing the PKA/CREB/BDNF signaling and inhibiting the expression of BDNF.

In summary, our current study firstly identified the rapid antidepressant effects of GJ-IFM in CUMS mice with the time course of changes in the endurance of these effects, which were consistent with acute administration. We further studied *via* the PKA-CREB signaling pathway the underlying mechanisms of GJ-IFM in the fast and enduring antidepressant effects. It has also produced additional evidence indicating that GJ-IF plays a crucial role in GJ’s rapid and lasting antidepressant activity. Future study direction should focus on investigating the mechanism of persistent antidepressant action of GJ-IF, which may link to different brain circuits and synaptic protein change. In addition, another key problem to be addressed is to elucidate the synergistic action of monomers in GJ-IF or GJ to exert rapid and enduring antidepressant effects.

## Data Availability

The original contributions presented in the study are included in the article/Supplementary Material, Further inquiries can be directed to the corresponding author.

## Abbreviations

GJ, *Gardenia jasminoides* J.Ellis [Rubiaceae]; GJ-PE, petroleum ether of *Gardenia jasminoides* J.Ellis [Rubiaceae]; GJ-BO, n-butyl alcohol fraction of *Gardenia jasminoides* J.Ellis [Rubiaceae]; GJ-IF, iridoid fraction of *Gardenia jasminoides* J.Ellis [Rubiaceae]; GJ-IFH, the high dose of iridoid fraction of *Gardenia jasminoides* J.Ellis [Rubiaceae]; GJ-IFM, the medium dose of iridoid fraction of *Gardenia jasminoides* J.Ellis [Rubiaceae]; GJ-IFL, the low dose of iridoid fraction of *Gardenia jasminoides* J.Ellis [Rubiaceae]; TST, tail suspension test; FST, forced swimming test; NSFT, novelty suppressed-feeding test; OFT, open field test; SPT, sucrose preference test; BDNF, brain-derived neurotrophic factor; PKA, cyclic-AMP dependent protein kinase A; p-CREB, phosphorylated cyclic-AMP responsive element-binding protein; CREB, cAMP response element-binding protein; CUMS, chronic unpredictable mild stress; NMDA, N-methyl-d-aspartic acid; NMDAR, N-methyl-d-aspartic acid receptor; PFC, prefrontal cortex; mPFC, medial prefrontal cortex.
